# (*E*)-Isopropyl 3-(3,4-dihy­droxy­phen­yl)acrylate

**DOI:** 10.1107/S1600536810044272

**Published:** 2010-11-04

**Authors:** Xu-Ji Shen, Shi-Yu Liu, Pu Jia, Shi-Xiang Wang, Xiao-Hui Zheng

**Affiliations:** a College of Life Sciences, Northwest University, Xi’an 710069, People’s Republic of China; b Affiliated High School, Northwest University, Xi’an 710069, People’s Republic of China

## Abstract

In the title compound, C_12_H_14_O_4_, a derivative of caffeic acid [(*E*)-3-(3,4-dihy­droxy­phen­yl)-2-propenoic acid], an intra­molecular O—H⋯O hydrogen bond forms an *S*(5) ring. In the crystal, inter­molecular O—H⋯O hydrogen bonds link mol­ecules into chains propagating in [110].

## Related literature

For the properties of caffeate esters, see: Uwai *et al.* (2008 ▶); Buzzi *et al.* (2009 ▶); Calheiros *et al.*(2008 ▶); Xia *et al.*(2008 ▶). For the preparation of the title compound, see: Hu *et al.* (2006 ▶). For hydrogen-bond motifs, see: Bernstein *et al.* (1995 ▶).
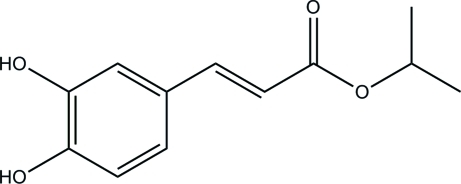


## Experimental

### 

#### Crystal data

C_12_H_14_O_4_
*M* *_r_* = 222.23
Triclinic, 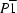

*a* = 5.8830 (14) Å
*b* = 9.644 (2) Å
*c* = 11.428 (3) Å
α = 65.690 (2)°
β = 89.370 (3)°
γ = 81.018 (3)°
*V* = 582.6 (2) Å^3^
*Z* = 2
Mo *K*α radiation
μ = 0.10 mm^−1^
*T* = 296 K
0.31 × 0.27 × 0.19 mm

#### Data collection

Bruker APEXII CCD diffractometer
2938 measured reflections
2042 independent reflections
1436 reflections with *I* > 2σ(*I*)
*R*_int_ = 0.015

#### Refinement

*R*[*F* ^2^ > 2σ(*F* ^2^)] = 0.045
*wR*(*F* ^2^) = 0.151
*S* = 1.05
2042 reflections
150 parameters
H-atom parameters constrained
Δρ_max_ = 0.16 e Å^−3^
Δρ_min_ = −0.13 e Å^−3^

### 

Data collection: *APEX2* (Bruker, 2009 ▶); cell refinement: *SAINT* (Bruker, 2009 ▶); data reduction: *SAINT*; program(s) used to solve structure: *SHELXS97* (Sheldrick, 2008 ▶); program(s) used to refine structure: *SHELXL97* (Sheldrick, 2008 ▶); molecular graphics: *SHELXTL* (Sheldrick, 2008 ▶); software used to prepare material for publication: *SHELXTL*.

## Supplementary Material

Crystal structure: contains datablocks I, global. DOI: 10.1107/S1600536810044272/lh5158sup1.cif

Structure factors: contains datablocks I. DOI: 10.1107/S1600536810044272/lh5158Isup2.hkl

Additional supplementary materials:  crystallographic information; 3D view; checkCIF report

## Figures and Tables

**Table 1:** Hydrogen-bond geometry (Å, °)

*D*—H⋯*A*	*D*—H	H⋯*A*	*D*⋯*A*	*D*—H⋯*A*
O3—H3⋯O2^i^	0.82	1.92	2.725 (2)	169
O4—H4⋯O3^ii^	0.82	2.09	2.792 (2)	143
O4—H4⋯O3	0.82	2.28	2.721 (2)	114

Symmetry codes: (i) 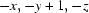; (ii) 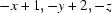.

**Fig. 1.**
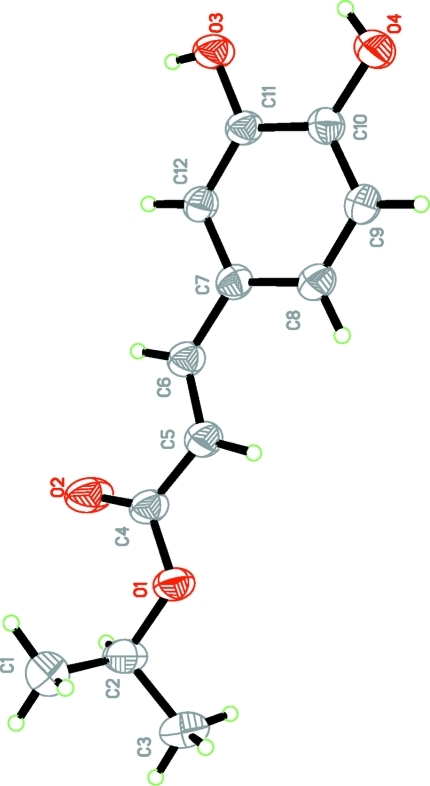
The molecular structure of (I) with the atom numbering scheme, showing displacement ellipsoids at the 30% probability level.

**Fig. 2.**
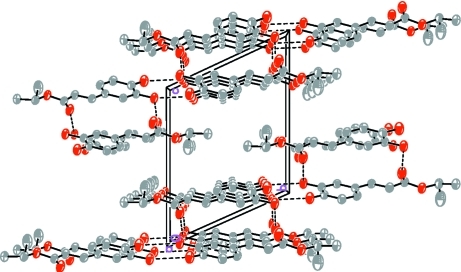
The packing of (I), viewed along the a axis. The dashed lines show the donor-acceptor distances of O—H···O hydrogen bonds. H atoms are not shown.

## supplementary crystallographic information

### Comment

Caffeate esters have been shown to have, an inhibitory effect on lipopolysaccharide-induced nitric oxide production, antinociceptive properties, and anticancer activity (Uwai *et al.*, 2008; Buzzi *et al.*, 2009; Calheiros *et al.*, 2008; Xia *et al.*, 2008).

The molecular structure of the title compound (I) is shown in Fig. 1. An intramolecular O-H···O hydrogen bond forms an S(5) ring (Bernstein *et al.*, 1995). In the crystal structure, intermolecular O—H···O hydrogen bonds link molecules into one-dimensional chains along [110] (see Fig .2).

### Experimental

The synthesis follows the method of Hu *et al.* (2006). To a solution of 0.02 *M* caffeic acid in 120 ml of 2-propanol at room temperature, 0.2 *M* HCl in 2-propanol was added. After the solution had been allowed to stir and reflux for 16 h, the solvent was removed under reduced pressure. The residue was extracted with ethyl acetate three times and filtered. The filtrate was washed successively with dilute saturated aqueous NaHCO_3_ solution, saturated aqueous NaCl, dried over MgSO_4_, and evaporated. The crude products were purified by chromatography (SiO_2_; elution with petroleum ether-acetoacetate, 6:1 *v*/*v*). Yield 80%. X-ray quality crystals were grown from a solution of the title compound in acetone and toluene at room temperature. Spectroscopic analysis: IR(KBr, χm^-1^): 3464, 3310, 2973, 2926, 1675, 1629, 1599, 1529, 1370, 1276; ^1^H NMR (DMSO, δ, p.p.m.): 9.606 (s, 1 H), 9.146 (s, 1 H), 7.424—7.464 (d, 1 H), 7.029 (s, 1 H), 6.990—7.011 (d, 1 H), 6.742—6.762 (d, 1 H), 6.246—6.206(d, 1 H), 4.951—5.013(m, 1 H), 1.245 (s, 3 H), 1.229 (s, 3 H).

### Refinement

H atoms were placed in calculated positions with O—H = 0.82Å and C—H = 0.93–0.96 Å with U_iso_(H) = 1.2U_eq_(C) or 1.5U_eq_(C_methyl_, O).

### Crystal data

**Table:**

C_12_H_14_O_4_	*Z* = 2
*M**_r_* = 222.23	*F*(000) = 236
Triclinic, *P*1	*D*_x_ = 1.267 Mg m^−^^3^
Hall symbol: -P 1	Melting point: 415 K
*a* = 5.8830 (14) Å	Mo *K*α radiation, λ = 0.71073 Å
*b* = 9.644 (2) Å	Cell parameters from 966 reflections
*c* = 11.428 (3) Å	θ = 2.4–26.5°
α = 65.690 (2)°	µ = 0.10 mm^−^^1^
β = 89.370 (3)°	*T* = 296 K
γ = 81.018 (3)°	Block, colorless
*V* = 582.6 (2) Å^3^	0.31 × 0.27 × 0.19 mm

### Data collection

**Table:**

Bruker APEXII CCD diffractometer	1436 reflections with *I* > 2σ(*I*)
Radiation source: fine-focus sealed tube	*R*_int_ = 0.015
graphite	θ_max_ = 25.1°, θ_min_ = 2.0°
φ and ω scans	*h* = −7→4
2938 measured reflections	*k* = −11→11
2042 independent reflections	*l* = −13→13

### Refinement

**Table:**

Refinement on *F*^2^	Secondary atom site location: difference Fourier map
Least-squares matrix: full	Hydrogen site location: inferred from neighbouring sites
*R*[*F*^2^ > 2σ(*F*^2^)] = 0.045	H-atom parameters constrained
*wR*(*F*^2^) = 0.151	*w* = 1/[σ^2^(*F*_o_^2^) + (0.0822*P*)^2^ + 0.0498*P*] where *P* = (*F*_o_^2^ + 2*F*_c_^2^)/3
*S* = 1.05	(Δ/σ)_max_ < 0.001
2042 reflections	Δρ_max_ = 0.16 e Å^−^^3^
150 parameters	Δρ_min_ = −0.13 e Å^−^^3^
0 restraints	Extinction correction: *SHELXL97* (Sheldrick, 2008), Fc^*^=kFc[1+0.001xFc^2^λ^3^/sin(2θ)]^-1/4^
Primary atom site location: structure-invariant direct methods	Extinction coefficient: 0.059 (11)

### Special details

**Table:**

Geometry. All e.s.d.'s (except the e.s.d. in the dihedral angle between two l.s. planes) are estimated using the full covariance matrix. The cell e.s.d.'s are taken into account individually in the estimation of e.s.d.'s in distances, angles and torsion angles; correlations between e.s.d.'s in cell parameters are only used when they are defined by crystal symmetry. An approximate (isotropic) treatment of cell e.s.d.'s is used for estimating e.s.d.'s involving l.s. planes.
Refinement. Refinement of *F*^2^ against ALL reflections. The weighted *R*-factor *wR* and goodness of fit *S* are based on *F*^2^, conventional *R*-factors *R* are based on *F*, with *F* set to zero for negative *F*^2^. The threshold expression of *F*^2^ > σ(*F*^2^) is used only for calculating *R*-factors(gt) *etc*. and is not relevant to the choice of reflections for refinement. *R*-factors based on *F*^2^ are statistically about twice as large as those based on *F*, and *R*- factors based on ALL data will be even larger.

### Fractional atomic coordinates and isotropic or equivalent isotropic displacement parameters (Å^2^)

**Table:**

	*x*	*y*	*z*	*U*_iso_*/*U*_eq_	
O1	0.2370 (2)	−0.06896 (13)	0.33802 (11)	0.0681 (4)	
O2	−0.0059 (3)	0.11022 (15)	0.18106 (14)	0.0914 (6)	
O3	0.3441 (2)	0.87422 (13)	−0.01294 (13)	0.0716 (4)	
H3	0.2302	0.8794	−0.0562	0.107*	
O4	0.7644 (2)	0.80457 (15)	0.11324 (14)	0.0808 (5)	
H4	0.6907	0.8884	0.0653	0.121*	
C1	−0.0940 (5)	−0.1810 (3)	0.4336 (3)	0.1171 (10)	
H1A	−0.0341	−0.1952	0.5163	0.176*	
H1B	−0.1815	−0.2610	0.4439	0.176*	
H1C	−0.1918	−0.0823	0.3940	0.176*	
C2	0.1019 (4)	−0.1880 (2)	0.34997 (19)	0.0718 (6)	
H2	0.0411	−0.1707	0.2647	0.086*	
C3	0.2645 (4)	−0.3381 (2)	0.4062 (2)	0.0847 (7)	
H3A	0.3904	−0.3349	0.3515	0.127*	
H3B	0.1847	−0.4205	0.4132	0.127*	
H3C	0.3228	−0.3552	0.4900	0.127*	
C4	0.1664 (3)	0.0739 (2)	0.25094 (17)	0.0612 (5)	
C5	0.3170 (3)	0.1807 (2)	0.25085 (17)	0.0631 (5)	
H5	0.4498	0.1430	0.3053	0.076*	
C6	0.2693 (3)	0.3291 (2)	0.17549 (16)	0.0585 (5)	
H6	0.1344	0.3605	0.1232	0.070*	
C7	0.4000 (3)	0.45108 (19)	0.16300 (15)	0.0539 (5)	
C8	0.6149 (3)	0.4221 (2)	0.22568 (17)	0.0606 (5)	
H8	0.6797	0.3213	0.2795	0.073*	
C9	0.7324 (3)	0.5406 (2)	0.20894 (17)	0.0639 (5)	
H9	0.8752	0.5194	0.2522	0.077*	
C10	0.6403 (3)	0.69140 (19)	0.12826 (16)	0.0582 (5)	
C11	0.4264 (3)	0.72247 (19)	0.06532 (15)	0.0550 (5)	
C12	0.3083 (3)	0.60347 (18)	0.08283 (15)	0.0550 (5)	
H12	0.1645	0.6252	0.0403	0.066*	

### Atomic displacement parameters (Å^2^)

**Table:**

	*U*^11^	*U*^22^	*U*^33^	*U*^12^	*U*^13^	*U*^23^
O1	0.0731 (9)	0.0470 (7)	0.0691 (8)	−0.0095 (6)	−0.0157 (6)	−0.0089 (6)
O2	0.1011 (12)	0.0557 (8)	0.0969 (10)	−0.0118 (8)	−0.0444 (9)	−0.0104 (8)
O3	0.0749 (9)	0.0457 (7)	0.0806 (9)	−0.0074 (6)	−0.0259 (7)	−0.0128 (6)
O4	0.0724 (9)	0.0577 (8)	0.1017 (11)	−0.0153 (7)	−0.0234 (8)	−0.0204 (8)
C1	0.0813 (17)	0.0802 (16)	0.167 (3)	−0.0154 (13)	0.0211 (17)	−0.0286 (17)
C2	0.0847 (14)	0.0521 (11)	0.0697 (12)	−0.0175 (10)	−0.0131 (10)	−0.0139 (9)
C3	0.1079 (18)	0.0533 (11)	0.0819 (14)	−0.0073 (11)	0.0031 (12)	−0.0192 (10)
C4	0.0665 (12)	0.0488 (10)	0.0578 (10)	−0.0044 (9)	−0.0117 (9)	−0.0132 (8)
C5	0.0617 (11)	0.0536 (11)	0.0644 (11)	−0.0074 (9)	−0.0098 (9)	−0.0153 (9)
C6	0.0622 (11)	0.0515 (10)	0.0549 (10)	−0.0062 (8)	−0.0053 (8)	−0.0161 (8)
C7	0.0561 (10)	0.0501 (10)	0.0515 (9)	−0.0055 (8)	−0.0005 (7)	−0.0180 (8)
C8	0.0613 (11)	0.0503 (10)	0.0593 (10)	−0.0018 (8)	−0.0065 (8)	−0.0143 (8)
C9	0.0550 (10)	0.0594 (11)	0.0686 (11)	−0.0038 (9)	−0.0117 (9)	−0.0196 (9)
C10	0.0574 (11)	0.0533 (10)	0.0610 (10)	−0.0099 (8)	−0.0040 (8)	−0.0204 (8)
C11	0.0593 (10)	0.0459 (9)	0.0537 (9)	−0.0051 (8)	−0.0066 (8)	−0.0157 (8)
C12	0.0539 (10)	0.0517 (10)	0.0540 (9)	−0.0049 (8)	−0.0072 (8)	−0.0176 (8)

### Geometric parameters (Å, °)

**Table:**

O1—C4	1.327 (2)	C3—H3C	0.9600
O1—C2	1.457 (2)	C4—C5	1.459 (3)
O2—C4	1.210 (2)	C5—C6	1.317 (2)
O3—C11	1.3735 (19)	C5—H5	0.9300
O3—H3	0.8200	C6—C7	1.462 (2)
O4—C10	1.358 (2)	C6—H6	0.9300
O4—H4	0.8200	C7—C8	1.391 (3)
C1—C2	1.500 (3)	C7—C12	1.395 (2)
C1—H1A	0.9600	C8—C9	1.372 (3)
C1—H1B	0.9600	C8—H8	0.9300
C1—H1C	0.9600	C9—C10	1.386 (2)
C2—C3	1.496 (3)	C9—H9	0.9300
C2—H2	0.9800	C10—C11	1.384 (2)
C3—H3A	0.9600	C11—C12	1.377 (2)
C3—H3B	0.9600	C12—H12	0.9300
			
C4—O1—C2	118.69 (14)	C6—C5—C4	121.51 (17)
C11—O3—H3	109.5	C6—C5—H5	119.2
C10—O4—H4	109.5	C4—C5—H5	119.2
C2—C1—H1A	109.5	C5—C6—C7	128.67 (18)
C2—C1—H1B	109.5	C5—C6—H6	115.7
H1A—C1—H1B	109.5	C7—C6—H6	115.7
C2—C1—H1C	109.5	C8—C7—C12	118.10 (16)
H1A—C1—H1C	109.5	C8—C7—C6	122.98 (16)
H1B—C1—H1C	109.5	C12—C7—C6	118.91 (16)
O1—C2—C3	106.18 (17)	C9—C8—C7	120.69 (17)
O1—C2—C1	108.92 (17)	C9—C8—H8	119.7
C3—C2—C1	112.69 (18)	C7—C8—H8	119.7
O1—C2—H2	109.7	C8—C9—C10	120.69 (17)
C3—C2—H2	109.7	C8—C9—H9	119.7
C1—C2—H2	109.7	C10—C9—H9	119.7
C2—C3—H3A	109.5	O4—C10—C11	122.00 (15)
C2—C3—H3B	109.5	O4—C10—C9	118.59 (16)
H3A—C3—H3B	109.5	C11—C10—C9	119.41 (16)
C2—C3—H3C	109.5	O3—C11—C12	123.54 (15)
H3A—C3—H3C	109.5	O3—C11—C10	116.67 (15)
H3B—C3—H3C	109.5	C12—C11—C10	119.79 (15)
O2—C4—O1	123.04 (17)	C11—C12—C7	121.31 (16)
O2—C4—C5	124.39 (16)	C11—C12—H12	119.3
O1—C4—C5	112.57 (15)	C7—C12—H12	119.3
			
C4—O1—C2—C3	155.38 (16)	C7—C8—C9—C10	−0.6 (3)
C4—O1—C2—C1	−83.0 (2)	C8—C9—C10—O4	−179.05 (16)
C2—O1—C4—O2	0.3 (3)	C8—C9—C10—C11	0.8 (3)
C2—O1—C4—C5	179.43 (15)	O4—C10—C11—O3	−0.4 (3)
O2—C4—C5—C6	2.5 (3)	C9—C10—C11—O3	179.78 (16)
O1—C4—C5—C6	−176.64 (16)	O4—C10—C11—C12	179.41 (17)
C4—C5—C6—C7	−179.87 (16)	C9—C10—C11—C12	−0.4 (3)
C5—C6—C7—C8	5.7 (3)	O3—C11—C12—C7	179.71 (14)
C5—C6—C7—C12	−175.59 (17)	C10—C11—C12—C7	−0.1 (3)
C12—C7—C8—C9	0.1 (3)	C8—C7—C12—C11	0.2 (3)
C6—C7—C8—C9	178.84 (17)	C6—C7—C12—C11	−178.53 (15)

### Hydrogen-bond geometry (Å, °)

**Table:**

*D*—H···*A*	*D*—H	H···*A*	*D*···*A*	*D*—H···*A*
O3—H3···O2^i^	0.82	1.92	2.725 (2)	169.
O4—H4···O3^ii^	0.82	2.09	2.792 (2)	143.
O4—H4···O3	0.82	2.28	2.721 (2)	114.

Symmetry codes: (i) −*x*, −*y*+1, −*z*; (ii) −*x*+1, −*y*+2, −*z*.
